# Nomogram predictive model for in-hospital mortality risk in elderly ICU patients with urosepsis

**DOI:** 10.1186/s12879-024-09319-8

**Published:** 2024-04-26

**Authors:** Jian Wei, Ruiyuan Liang, Siying Liu, Wanguo Dong, Jian Gao, Tianfeng Hua, Wenyan Xiao, Hui Li, Huaqing Zhu, Juanjuan Hu, Shuang Cao, Yu Liu, Jun Lyu, Min Yang

**Affiliations:** 1 The Second Department of Critical Care Medicine, The Second Affiliated Hospital of Anhui Medical University, 678 Furong Road, 230601 Hefei, Anhui Province China; 2 Laboratory of Cardiopulmonary Resuscitation and Critical Care, The Second Affiliated Hospital of Anhui Medical University, 678 Furong Road, 230601 Hefei, Anhui Province China; 3 Key Laboratory of Intelligent Computing & Signal Processing, Ministry of Education, Anhui University, 111 Jiulong Road, 230601 Hefei, Anhui Province China; 4 School of Integrated Circuits, Anhui University, 111 Jiulong Road, 230601 Hefei, Anhui Province China; 5 Laboratory of Molecular, Biology and Department of Biochemistry, Anhui Medical University, 81 Meishan Road, 230022 Hefei, Anhui Province China; 6 Department of Clinical Research, The First Affiliated Hospital of Jinan University, 613 West Huangpu Avenue, Tianhe District, 510630 Guangzhou, Guangdong Province China

**Keywords:** Urinary tract infection, Urosepsis, Nomogram, MIMIC-IV

## Abstract

**Background:**

Urinary tract infection (UTI) is a common cause of sepsis. Elderly patients with urosepsis in intensive care unit (ICU) have more severe conditions and higher mortality rates owing to factors such as advanced age, immunosenescence, and persistent host inflammatory responses. However, comprehensive studies on nomograms to predict the in-hospital mortality risk in elderly patients with urosepsis are lacking. This study aimed to construct a nomogram predictive model to accurately assess the prognosis of elderly patients with urosepsis and provide therapeutic recommendations.

**Methods:**

Data of elderly patients with urosepsis were extracted from the Medical Information Mart for Intensive Care (MIMIC) IV 2.2 database. Patients were randomly divided into training and validation cohorts. A predictive nomogram model was constructed from the training set using logistic regression analysis, followed by internal validation and sensitivity analysis.

**Results:**

This study included 1,251 patients. LASSO regression analysis revealed that the Glasgow Coma Scale (GCS) score, red cell distribution width (RDW), white blood count (WBC), and invasive ventilation were independent risk factors identified from a total of 43 variables studied. We then created and verified a nomogram. The area under the receiver operating characteristic curve (AUC), net reclassification improvement (NRI), integrated discrimination improvement (IDI), and decision curve analysis (DCA) of the nomogram were superior to those of the traditional SAPS-II, APACHE-II, and SOFA scoring systems. The Hosmer-Lemeshow test results and calibration curves suggested good nomogram calibration. The IDI and NRI values showed that our nomogram scoring tool performed better than the other scoring systems. The DCA curves showed good clinical applicability of the nomogram.

**Conclusions:**

The nomogram constructed in this study is a convenient tool for accurately predicting in-hospital mortality in elderly patients with urosepsis in ICU. Improving the treatment strategies for factors related to the model could improve the in-hospital survival rates of these patients.

## Background

Sepsis, characterized by a detrimental host response to various severe infections, is one of the most critical medical conditions worldwide, resulting in high mortality rates among patients in intensive care unit (ICU) [1, 2]. Sepsis affects more than 50 million people worldwide and is associated with more than 10 million deaths annually. Timely detection and management of sepsis can improve outcomes [3]. However, early clinical symptoms of sepsis are nonspecific, and the disease can rapidly progress and worsen, with currently available treatments having limited effectiveness [4]. This challenge is further exacerbated in the elderly due to factors such as increased age, immunosenescence, and continuous host inflammatory responses [5]. Studies have identified age as an important risk factor for mortality in patients with sepsis, with over 60% of elderly patients (aged > 65 years) at risk of developing sepsis, and more than 75% of these cases resulting in death from the condition [6–8]. Aging is associated with various physiological changes, including a weakened immune response, which reduces the body’s ability to effectively resist infections. Additionally, even in the absence of infection, an increase in inflammatory activity (referred to as “inflamm-aging”) can lead to an exacerbated state of inflammation, thereby intensifying the severity of sepsis [9].

Sepsis is a complex condition caused by several factors affecting the function of different organs. The most lethal cases of sepsis primarily stem from lower respiratory tract infections; however, urinary tract infection (UTI) is a rapidly increasing cause of sepsis. Among patients over 65, nearly 30% of sepsis cases may originate from UTI. UTI is the second most common cause of hospitalization among the elderly, after pneumonia [10]. Urosepsis, a severe condition caused by severe UTI that leads to organ failure, is an important cause of sepsis [11]. Approximately 30% of the sepsis cases in the United States are attributable to UTI [12]. Gharbi et al. [13] found that owing to physiological tendencies, such as the decline of the immune system, as well as the presence of chronic diseases that alter immune function, such as diabetes and chronic kidney failure, elderly populations diagnosed with urosepsis have more severe conditions, and these diseases are associated with higher mortality rates. However, there are currently no effective scales for assessing the prognosis and status of elderly patients with urosepsis, leading to a delay in initiating precise treatment.

Recently, significant advancements have been made in sepsis management, including the development of rapid diagnostic tools. These advances have significantly reduced the time required for pathogen identification, enabling timely and targeted treatment strategies. Although current scoring systems such as the Acute Physiology and Chronic Health Evaluation-II (APACHE-II), the Simplified Acute Physiology Score-II (SAPS-II), and Sequential Organ Failure Assessment (SOFA) are somewhat useful in assessing patient conditions, these scoring systems also have limitations, such as their complexity and their primary focus being on assessing organ physiological functions, which makes their operation cumbersome [14–17]. These scoring systems were originally designed to predict mortality in the general ICU population but not specifically in elderly patients with urosepsis. The prognostic assessment and clinical practice of elderly patients with urosepsis are not well guided by these scores, which are not sufficiently sensitive or relevant to guide treatment decisions. Boonmee et al. [18] also identified that these scoring systems are often used to predict mortality rates following sepsis in emergency departments. However, the accuracy of these standards may be reduced because of the different clinical presentations in elderly patients. Given the absence of effective scales for predicting outcomes in elderly individuals with urosepsis, this study aims to investigate the risk factors for in-hospital mortality in this population, construct a nomogram predictive model, and compare it with the SAPS-II system to accurately assess patient status, predict prognosis outcomes, and offer treatment recommendations for elderly patients with urosepsis.

## Methods

### Data sources

A study was carried out using the Medical Information Mart for Intensive Care (MIMIC) IV database (version 2.2), which includes two inpatient database systems, a tailored hospital-wide electronic health record and a specialized clinical information system for the ICU, covering the period from 2008 to 2019 [19]. The data of the patients in this database has been de-identified, eliminating the necessity of obtaining informed consent for this research. After participating in a sequence of classes provided by the National Institutes of Health, the researchers were granted permission to access the MIMIC-IV 2.2 database upon successful completion of the mandatory evaluations (certificate number 55,437,665).

### Study population

The following criteria were used for inclusion: (1) initial admission to the ICU, (2) diagnosis of sepsis and UTI, and (3) age ≥ 65 years. Participants were excluded if they had a SOFA score of < 2 or if they stayed in the ICU for less than 24 h.

**Fig. 1:**
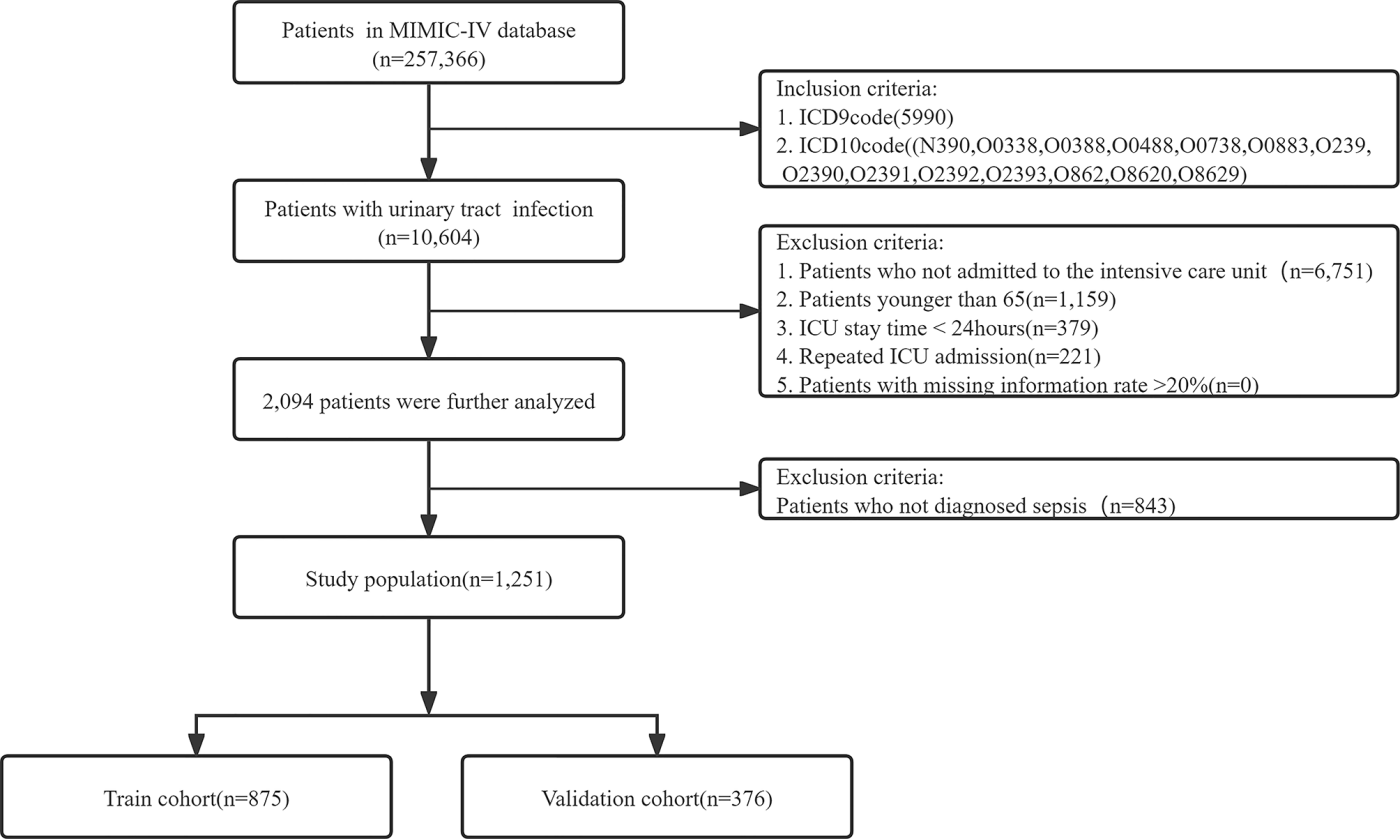
Flow chart of patient selection

Sepsis is diagnosed using the 2016 revision of the sepsis-3 [20] criteria. The criteria include a life-threatening infection and a sudden elevation in the SOFA score (SOFA ≥ 2). Patients diagnosed with UTI were extracted from the MIMIC-IV database using the International Classification of Diseases Ninth Revision (ICD-9) code 5990 and Tenth Revision (ICD-10) codes N390, O0338, O0388, O0488, O0738, O0883, O239, O2390, O2391, O2392, O2393, O862, O8620, and O8629. Patients under 65 years of age were excluded. The process is depicted in Fig. 1.

### Data extraction

We used Structured Query Language in Navicat Premium version 15.0.23 to extract the necessary data from the MIMIC-IV database, which underwent rigorous validation and filtering in accordance with the best practices in scientific computing. A multidisciplinary group of physicians and researchers performed the code evaluations to ensure data reliability. The extracted information included: (1) Demographics: age, sex, and race, which helped analyze disease distribution and outcomes across different populations, providing a foundation for assessing the impact of diseases. (2) Comorbidities: urolithiasis, malignant cancer, congestive heart failure, diabetes, hypertension, severe liver disease, obesity, chronic pulmonary disease, and renal failure. Data on comorbid conditions were crucial for evaluating the overall health status and prognosis of patients, affecting treatment choices and outcomes. (3) Laboratory tests: white blood cell (WBC), neutrophils, lymphocytes, hemoglobin, hematocrit, platelets, red cell distribution width (RDW), creatinine, glucose, albumin, sodium, calcium, chloride, potassium, pH, pO2, pCO2, base excess, lactate, urinary white blood cells, urinary red blood cells, urine blood, urine ketone, urine protein, and total input in the first 24 h of ICU admission. These indicators reflected the physiological and metabolic state of patients, essential for the diagnosis and monitoring of diseases. (4) Disease severity scores: Glasgow Coma Scale (GCS), SAPS-II, APACHE-II, SOFA. These scoring systems were used to assess disease severity at the time of ICU admission, which was significant for predicting hospital outcomes. (5) Treatments: intravenous antibiotics, urinary catheter insertion, and urological surgery; the detailed recording of treatment measures were crucial for analyzing the impact of specific interventions on the disease process and outcomes. (6) Outcome: in-hospital mortality.

### Statistical analysis

Variables with more than 20% missing data were excluded from the analysis. For the remaining dataset, we used the “mice” package in R software to fill in missing values.

Elderly patients with urosepsis included in this study were randomly allocated to a training cohort and a validation cohort in a ratio of 7:3 (random seed number: 1). The training cohort was used for the development of a nomogram, whereas the validation cohort was used for internal validation. Categorical variables are expressed as frequencies and percentages, and group differences are analyzed using either the chi-square test or Fisher’s exact test. The normal distribution of continuous variables was assessed using the Shapiro-Wilk test. Continuous variables are expressed as means and standard deviations or medians and interquartile ranges.

In this study, we developed a nomogram to use to predict in-hospital mortality among older patients with urosepsis. Although advanced predictive techniques such as machine learning are available, they are not necessarily superior to traditional models for all clinical scenarios. Machine learning models require large datasets for training and may not provide the ease of interpretability offered by traditional models. In the context of our study, we chose to use a nomogram because of its straightforward interpretation and ease of use in clinical settings, and because nomograms are widely used and accepted in medical and clinical research, which facilitates their adoption by healthcare professionals. Rahmatinejad et al. [21] pointed out that machine learning models are not necessarily superior to traditional regression-based models in predicting in-hospital mortality in similar contexts, and noted that traditional models can achieve levels of accuracy similar to those of machine learning models.

To select predictors, we used the Least Absolute Shrinkage and Selection Operator (LASSO) regression analysis, and factors with non-zero coefficients were selected. The LASSO method was employed on the training cohort data to identify optimal predictors of current risk factors. These variables were initially used to screen risk factors.

A predictive model was developed through multivariable logistic regression analysis by, incorporating the features identified in the LASSO regression model. The significance of these characteristics was assessed using odds ratio (OR), along with the corresponding 95% confidence interval (CI) and *p*-values. By including all the selected features and assessing their statistical significance, a nomogram model for in-hospital mortality risk was established using predictors that demonstrated statistical significance.

Furthermore, we employed various techniques to validate our nomogram model. The receiver operating characteristic (ROC) curve was used to assess the discriminative performance of the nomogram compared with the SAPS-II, APACHE-II, and SOFA scoring systems. Calibration curves was used to measure the agreement between the predicted probabilities and the actual results. Additionally, decision curve analysis (DCA) was conducted to evaluate the clinical usefulness of the nomogram by examining the net benefit across different threshold probabilities. Moreover, net reclassification improvement (NRI) and integrated discrimination improvement (IDI) were used to assess the performance improvements of the nomogram compared with other scoring systems.

R software version 4.2.3 (The R Foundation for Statistical Computing, Vienna, Austria) and Stata version 17.0 (StataCorp, College Station, TX, USA) were used for statistical analyses. The R packages included compareGroups, glmnet, rms, mice, foreign, tidyverse, pROC, regplot, calibration, nricens, and rmda. Statistical significance was set at *P* < 0.05.

## Results

### Baseline characteristics

In our study of 1,251 elderly patients with urosepsis, 503 were male and 748 were female, with 235 patients dying in hospital and 1,016 surviving. Random allocation was used to assign patients to the training cohort consisting of 875 individuals and the validation cohort consisting of 376 individuals, at a ratio of 7:3. All patients underwent the relevant examinations. The median age of the training and validation groups were 79.0 [73.0, 86.0] years and 79.5 [73.0, 87.0] years, respectively. In the training group, urolithiasis was found in 1.71% of cases, compared to 3.99% in the validation group. The rates of renal disease were similar in both groups (34.3% and 34.6%, respectively). Diabetes was found in 35.2% and 39.6% of the participants in the training and validation groups, respectively. In both cohorts, the median SAPS-II score was 43.0 [36.0, 51.0], and the median SOFA score was 6.0 [4.0, 8.0]. The median APACHE II scores of the training and validation groups were 23.0 [19.0, 28.0] and 22.0 [18.0, 27.0], respectively. Laboratory tests revealed proteinuria in 67.9% and 64.4% of the training and validation cohorts. The remaining baseline characteristics of the patients in the two cohorts are listed in Table 1.

**Table 1:** Demographic and clinical characteristics of patients

Variable	ALL	Training cohort	Validation cohort
N	1251	875	376
Sex(%)			
Male	503 (40.2%)	363 (41.5%)	140 (37.2%)
Female	748 (59.8%)	512 (58.5%)	236 (62.8%)
Race(%)			
white	861 (68.8%)	609 (69.6%)	252 (67.0%)
black	133 (10.6%)	92 (10.5%)	41 (10.9%)
other	257 (20.5%)	174 (19.9%)	83 (22.1%)
Age	79.0 [73.0,86.0]	79.0 [73.0,86.0]	79.5 [73.0,87.0]
GCS	13.0 [8.00,14.0]	13.0 [9.00,14.0]	13.0 [8.00,14.0]
SAPS-II	43.0 [36.0,51.0]	43.0 [36.0,51.0]	43.0 [36.0,51.0]
APACHE-II	23.0[18.0,28.0]	23.0[19.0,28.0]	22.0[18.0,27.0]
SOFA	6.00[4.00,8.00]	6.00[4.00,8.00]	6.00[4.00,8.00]
Charlson	7.00 [6.00,9.00]	7.00 [6.00,9.00]	7.00 [6.00,9.00]
Comorbidities			
Urolithiasis(%)			
No	1221 (97.6%)	860 (98.3%)	361 (96.0%)
Yes	30 (2.40%)	15 (1.71%)	15 (3.99%)
Malignant cancer(%)			
No	1083 (86.6%)	748 (85.5%)	335 (89.1%)
Yes	168 (13.4%)	127 (14.5%)	41 (10.9%)
Congestive heart failure(%)			
No	723 (57.8%)	516 (59.0%)	207 (55.1%)
Yes	528 (42.2%)	359 (41.0%)	169 (44.9%)
Diabetes(%)			
No	794 (63.5%)	567 (64.8%)	227 (60.4%)
Yes	457 (36.5%)	308 (35.2%)	149 (39.6%)
Hypertension(%)			
No	1223 (97.8%)	858 (98.1%)	365 (97.1%)
Yes	28 (2.24%)	17 (1.94%)	11 (2.93%)
Severe liver disease(%)			
No	1203 (96.2%)	846 (96.7%)	357 (94.9%)
Yes	48 (3.84%)	29 (3.31%)	19 (5.05%)
Obesity(%)			
No	1119 (89.4%)	785 (89.7%)	334 (88.8%)
Yes	132 (10.6%)	90 (10.3%)	42 (11.2%)
Chronic pulmonary disease(%)			
No	949 (75.9%)	652 (74.5%)	297 (79.0%)
Yes	302 (24.1%)	223 (25.5%)	79 (21.0%)
Renal disease(%)			
No	821 (65.6%)	575 (65.7%)	246 (65.4%)
Yes	430 (34.4%)	300 (34.3%)	130 (34.6%)
Laboratory test			
WBC(K/uL)	13.5 [9.50,18.4]	13.4 [9.50,18.2]	13.6 [9.70,18.8]
Neutrophil(%)	75.2 [65.4,83.6]	75.2 [66.5,84.0]	74.8 [63.2,83.0]
Lymphocytes(%)	14.6 [8.00,23.1]	14.6 [7.80,22.5]	15.0 [8.80,24.3]
Hematocrit(g/dL)	29.8 [25.0,34.6]	29.8 [25.0,34.5]	29.7 [24.9,35.2]
Hemoglobin(g/dL)	9.40 [7.90,11.1]	9.40 [7.90,11.1]	9.50 [7.90,11.2]
Platelets(K/uL)	173 [121,233]	174 [121,230]	171 [121,237]
RDW(%)	15.5 [14.2,17.3]	15.5 [14.2,17.2]	15.5 [14.3,17.5]
Creatinine(mg/dL)	1.30 [0.90,2.00]	1.20 [0.90,2.00]	1.30 [0.90,1.90]
Glucose(mg/dL)	149 [118,200]	150 [121,202]	145 [115,198]
Albumin(g/dL)	3.60 [3.10,4.00]	3.60 [3.10,4.00]	3.60 [3.20,4.00]
Sodium(mmol/L)	140 [137,143]	141 [137,144]	140 [138,143]
Calcium(mg/dL)	8.20 [7.70,8.70]	8.20 [7.70,8.70]	8.30 [7.80,8.70]
Chloride(mmol/L)	105 [100,109]	105 [100,109]	105 [101,109]
Potassium(mmol/L)	4.50 [4.10,5.10]	4.50 [4.10,5.10]	4.50 [4.10,5.10]
Ph	7.40 [7.30,7.40]	7.40 [7.30,7.40]	7.40 [7.30,7.40]
Po2(mmHg)	70.0 [43.5,138]	70.0 [44.0,137]	70.0 [43.0,142]
Pco2(mmHg)	41.0 [35.0,47.0]	41.0 [35.0,47.0]	40.0 [34.8,47.0]
Base excess(mmol/L)	0.00 [-4.00,1.00]	0.00 [-4.00,1.00]	0.00 [-4.00,1.00]
Lac(mmol/L)	1.70 [1.20,2.50]	1.70 [1.20,2.50]	1.70 [1.30,2.40]
Urine RBC(#/hpf)	3.00 [1.00,12.0]	3.00 [1.00,12.0]	4.00 [1.00,12.0]
Urine WBC(#/hpf)	7.00 [2.00,32.0]	7.00 [2.00,34.5]	7.00 [2.00,27.2]
Input total(ml)	6410 [3260,12405]	6400 [3316,12100]	6460 [3200,13055]
Urine blood(%)			
Negative	647 (51.7%)	459 (52.5%)	188 (50.0%)
Positive	604 (48.3%)	416 (47.5%)	188 (50.0%)
Urine ketone(%)			
Negative	415 (33.2%)	305 (34.9%)	110 (29.3%)
Positive	836 (66.8%)	570 (65.1%)	266 (70.7%)
Urine protein(%)			
Negative	415 (33.2%)	281 (32.1%)	134 (35.6%)
Positive	836 (66.8%)	594 (67.9%)	242 (64.4%)
Urine catheter(%)			
No	908 (72.6%)	635 (72.6%)	273 (72.6%)
Yes	343 (27.4%)	240 (27.4%)	103 (27.4%)
Antibiotic(%)			
No	254 (20.3%)	172 (19.7%)	82 (21.8%)
Yes	997 (79.7%)	703 (80.3%)	294 (78.2%)
Invasive ventlation(%)			
No	756 (60.4%)	536 (61.3%)	220 (58.5%)
Yes	495 (39.6%)	339 (38.7%)	156 (41.5%)
Urologic surgery(%)			
No	1202 (96.1%)	360 (95.7%)	842 (96.2%)
Yes	49 (3.92%)	16 (4.26%)	33 (3.77%)
Outcome			
Mortality hospital(%)			
No	1016 (81.2%)	709 (81.0%)	307 (81.6%)
Yes	235 (18.8%)	166 (19.0%)	69 (18.4%)

Abbreviations: SAPS-II, Simplified Acute Physiologic Score-II; GCS, Glasgow Coma Scale; SOFA, Sequential Organ Failure Assessment; APACHE-II, Acute Physiology and Chronic Health Evaluation-II; WBC, white blood cell; RDW, red cell distribution width

### Predictive model construction

**Fig. 2:**
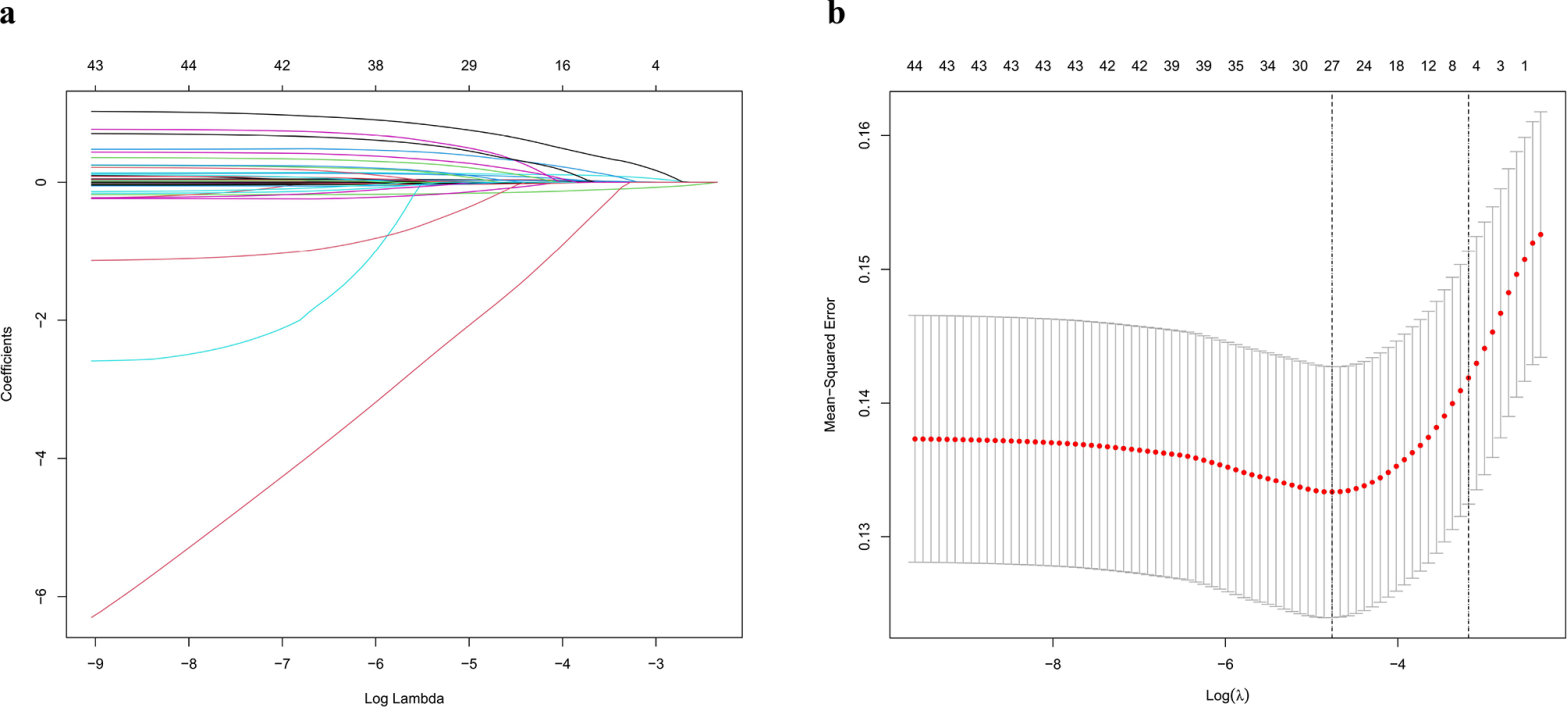
Variable selection using the LASSO model for binary logistic regression. (**a**) Coefficient paths of different variables using the LASSO model: four variables with nonzero coefficients were chosen by the optimal lambda. (**b**) Cross-validation plot with 1SE bounds using the LASSO model: the left and right dotted vertical lines represent the values of log (lambda. min) and log (lambda.1se), respectively. Following validation of the optimal parameter (lambda) in the LASSO model, we plotted the partial likelihood deviance (binomial deviance) curve versus log(lambda) and drew dotted vertical lines based on 1 standard error criteria. 10-fold cross-validation was conducted in the LASSO regression

**Fig. 3:**
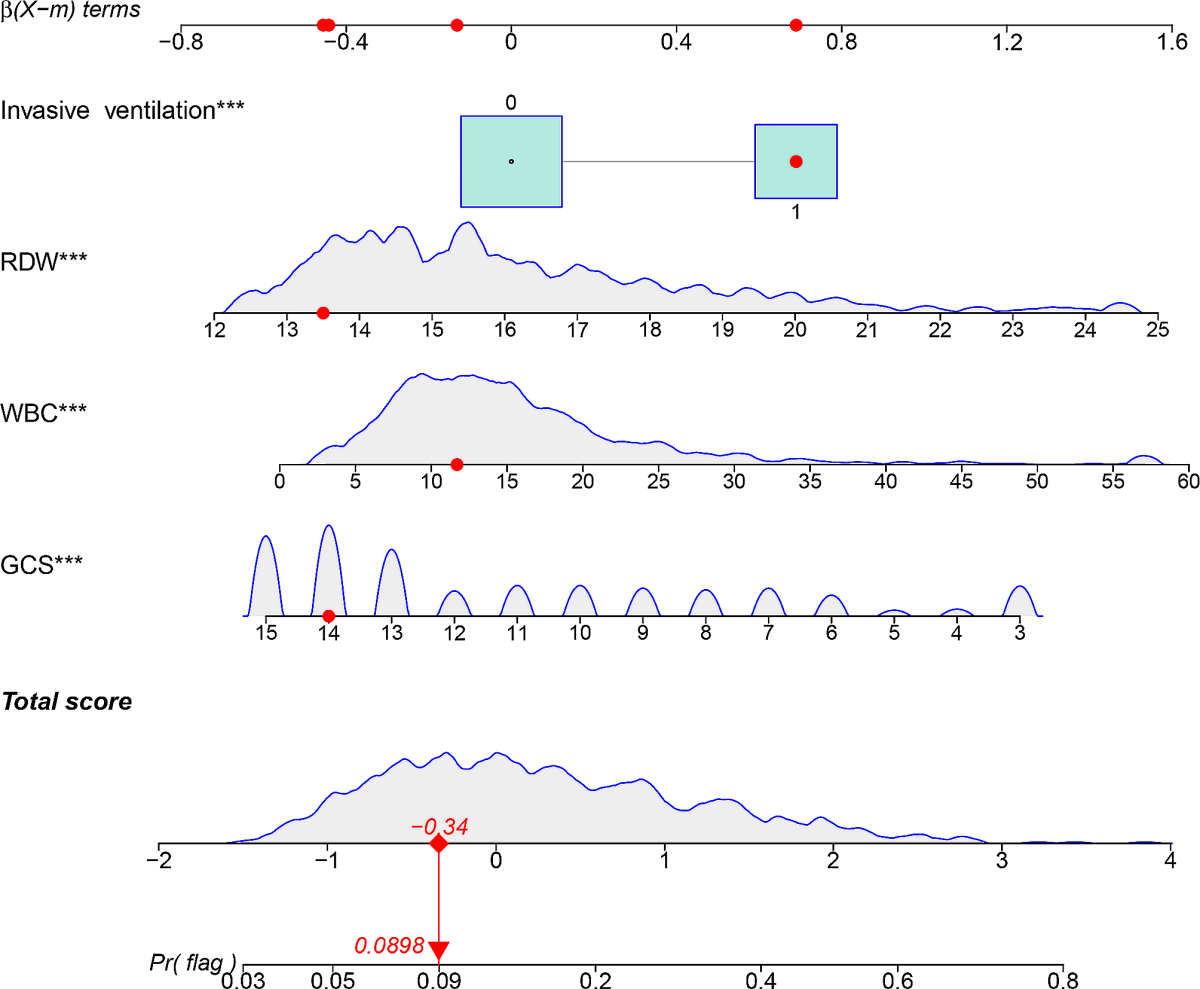
Nomogram model predicts in-hospital mortality in elderly patients with urosepsis. * represents *P* value < 0.05, and *** represents *P* value < 0.001

Predictive variables were chosen using LASSO regression analysis from the variables listed in Table 1, and a predictive model was developed using multivariable logistic regression analysis. The results showed that when the lambda value was selected as lambda. min (0.008544), 27 variables with non-zero coefficients were screened out (Fig. 2). When the lambda value was selected as lambda.1se (0.041544), four variables were identified as predictors in the predictive model: GCS, WBC, RDW, and invasive ventilation, all of which exhibited non-zero coefficients in the LASSO regression model (Fig. 2). The predictive model was presented as a nomogram to provide a quantitative estimation of the probability of in-hospital mortality in elderly patients with urosepsis (Fig. 3).

Table 2 shows the results of the logistic regression analyses of the four variables. As all predictors demonstrated statistically significant differences, suggesting their independence, they were included in the predictive model to construct an in-hospital mortality risk nomogram (Fig. 3). For example, using the nomogram model, a urosepsis patient with a GCS score of 14, WBC of 11.7mmHg, RDW of 13.5%, and undergoing invasive ventilation was estimated to have an 8.98% probability of in-hospital mortality (Fig. 3).

**Table 2:** Risk factors related to in-hospital mortality

characteristics	B	SE	OR	CI	Z	P
GCS	-0.152	0.025	0.86	0.82–0.90	-6.131	< 0.001
WBC	0.037	0.01	1.04	1.02–1.06	3.843	< 0.001
RDW	0.176	0.035	1.19	1.11–1.28	5.051	< 0.001
Invasive ventilation	0.690	0.193	1.99	1.37–2.91	3.575	< 0.001

Abbreviations: GCS, Glasgow Coma Scale; WBC, white blood cell; RDW, red cell distribution width; OR, odds ratio; CI, confidence interval.

### Nomogram validation

**Fig. 4:**
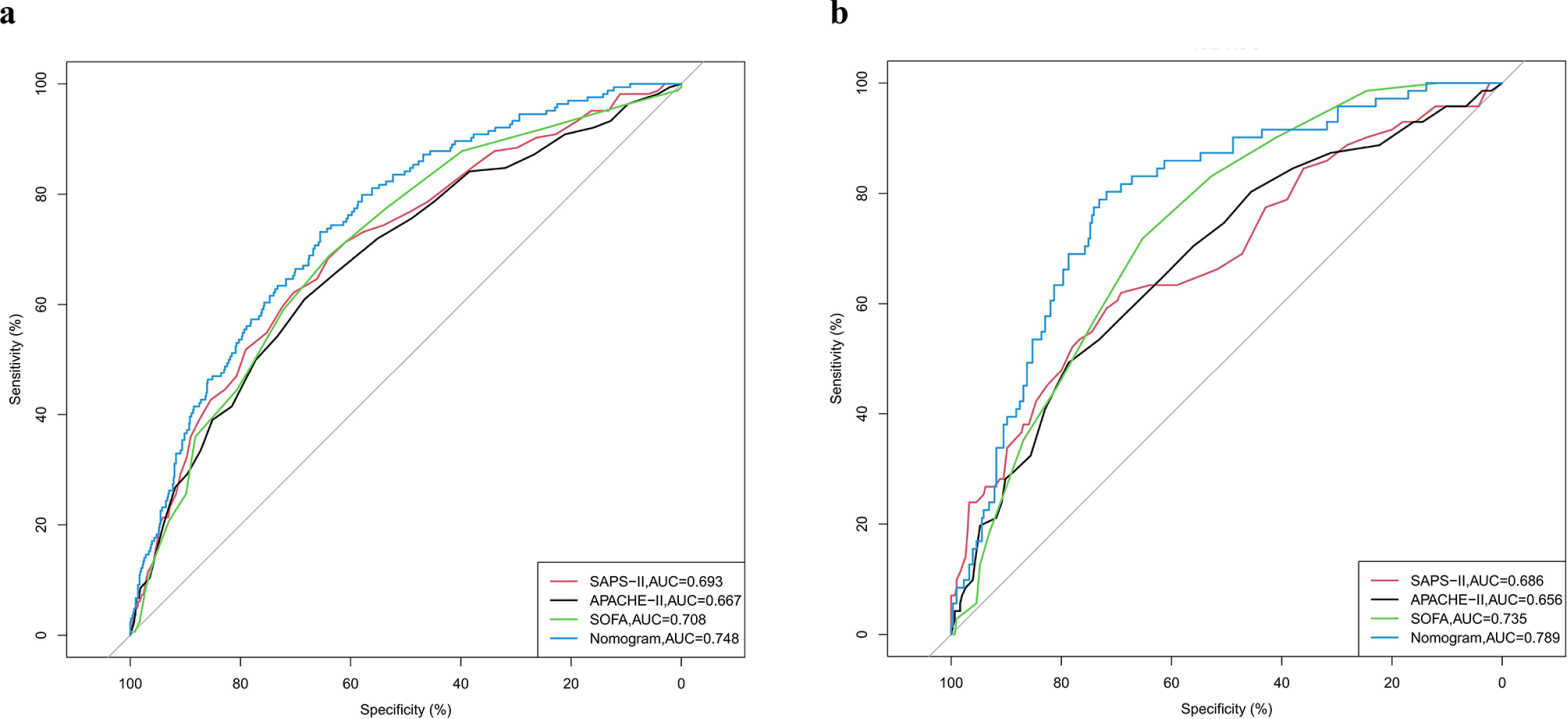
Receiver operating characteristic (ROC) curves for the SAPS-II model, APACHE-II model, SOFA model, and the nomogram. (**a**) Training set; (**b**) validation set

In this study, we conducted a comparative analysis of the predictive capabilities of our nomogram and the SAPS-II, APACHE-II, and SOFA scoring systems for in-hospital mortality among elderly patients with urosepsis. The results presented in Fig. 4 demonstrate that the area under the curve (AUC) values for the nomogram were 0.748 (95% CI 0.708–0.785) for the training cohort and 0.789 (95% CI 0.720–0.832) for the validation cohort, both of which outperformed the SAPS-II, APACHE-II, and SOFA scoring systems. In addition, the NRI and IDI metrics provided further evidence of the nomogram’s enhanced predictive accuracy. NRI values for the nomogram compared to the SAPS-II system were 0.125 (95% CI 0.047–0.203) and 0.266 (95% CI 0.155–0.376) in the training and validation cohorts, respectively (Table 3); the corresponding IDI values were 0.043 (95% CI 0.012–0.073), *P* < 0.001, and 0.078 (95% CI 0.028–0.129), *P* = 0.002, respectively (Table 3). Analysis comparing the nomogram with the APACHE II and SOFA scoring systems revealed trends similar to those observed for the SAPS-II system (Table 3). These findings suggest that the nomogram has better discriminative ability and outperforms the SAPS-II, APACHE II, and SOFA scoring systems.

**Table 3:** Predictive performances and validation of the nomogram

Predictive model	AUC	P	NRI	P	IDI	P
Training set						
Nomogram	0.748(0.708–0.785)					
SAPS-II	0.693(0.649–0.741)	< 0.001	0.125(0.047–0.203)	< 0.001	0.043(0.012–0.073)	< 0.001
APACHE-II	0.667(0.621–0.716)	< 0.001	0.057(0.018–0.096)	0.004	0.059(0.030–0.087)	< 0.001
SOFA	0.708(0.678–0.761)	< 0.001	0.138(0.044–0.232)	0.004	0.051(0.023–0.079)	< 0.001
Validation set						
Nomogram	0.789(0.720–0.832)					
SAPS-II	0.686(0.615–0.759)	< 0.001	0.266(0.155–0.376)	< 0.001	0.078(0.028–0.129)	0.002
APACHE-II	0.656(0.588–0.732)	< 0.001	0.096(0.021–0.171)	0.012	0.107(0.057–0.158)	< 0.001
SOFA	0.735(0.680–0.794)	< 0.001	0.142(0.007–0.276)	0.038	0.088(0.039–0.138)	< 0.001

Abbreviations: AUC, area under the receiver operating characteristic curve; NRI, net reclassification improvement; IDI, integrated discrimination improvement; SAPS-II, Simplified Acute Physiologic Score-II; SOFA, Sequential Organ Failure Assessment; APACHE-II, Acute Physiology and Chronic Health Evaluation-II.

**Fig. 5:**
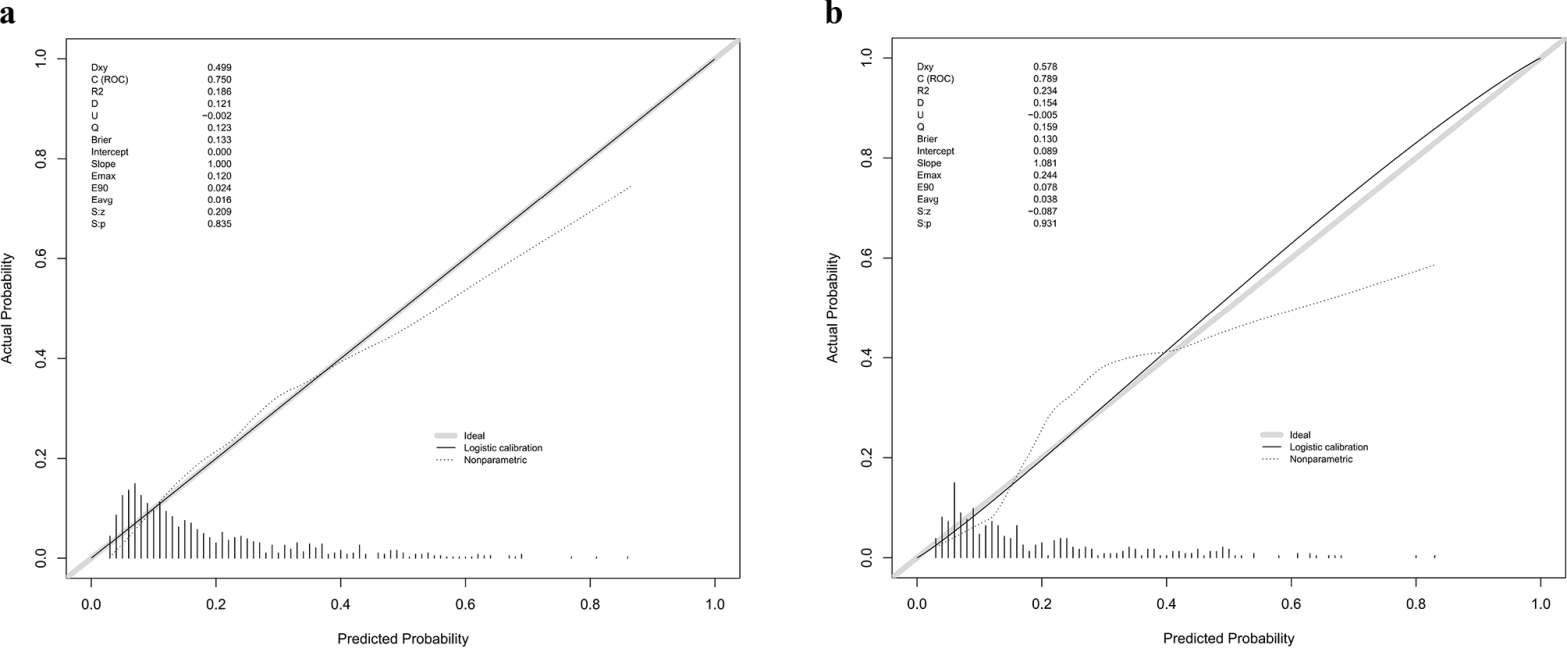
Calibration curves for the nomogram. (**a**) Training set; (**b**) validation set

**Fig. 6:**
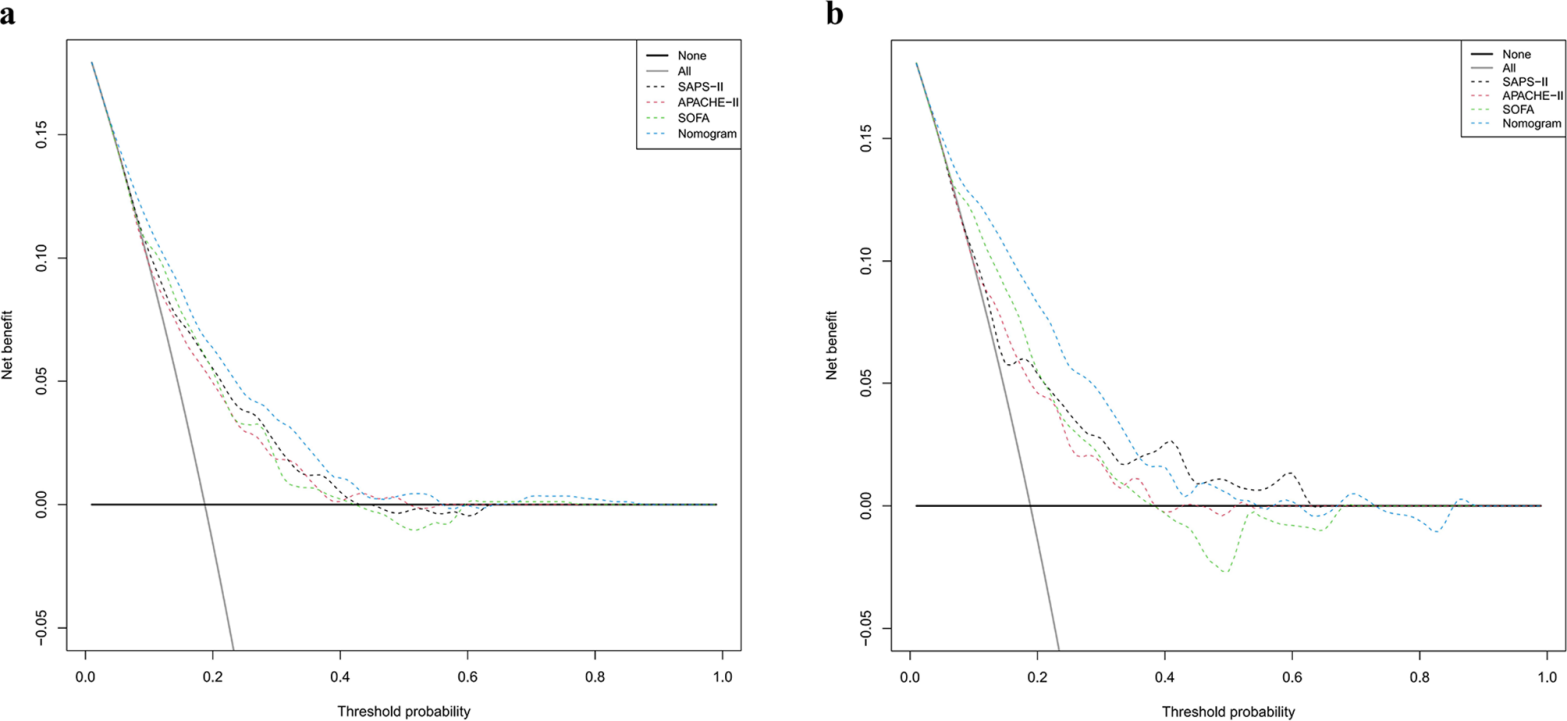
DCA curve for the SAPS-II model, APACHE-II model, SOFA model, and the nomogram. (**a**) Training set; (**b**) validation set

The calibration plot provides a more accurate reflection of whether the actual results for each nomogram match the predicted results. The nearly diagonal calibration curves of the nomogram for both the training and validation cohorts are displayed in Fig. 5. The Hosmer-Lemeshow test was no statistical significance, with the training cohort having a χ^2^ value of 7.899 and a *P* value of 0.544, and the validation cohort having a χ^2^ value of 12.330 and a *P* value of 0.195, which confirmed that the nomogram’s fit was appropriate. This indicates that the predicted probabilities of in-hospital mortality closely matched the observed outcomes, thus enhancing the reliability of the model. DCA was used to assess the clinical value of a model by comparing the standardized net benefit with the risk threshold probability [22]. The practicality of the nomogram was demonstrated using the DCA curves (Fig. 6). Our nomogram led to a higher overall advantage in clinical interventions compared with the other scoring systems when the threshold probability was between 0.1 and 0.6. This indicates that the use of a nomogram could potentially lead to better clinical outcomes by accurately identifying patients who would benefit from specific interventions.

## Discussion

This study effectively created and validated a predictive nomogram model to assess the likelihood of in-hospital mortality in elderly ICU patients with urosepsis. By incorporating significant variables such as GCS, WBC, RDW, and invasive ventilation, our model offers a straightforward and statistically sound method for predicting in-hospital mortality. The nomogram demonstrated strong performance, as evidenced by metrics, including AUC, NRI, IDI, and DCA. Compared with other scoring systems, our clinical model exhibited superior predictive and discriminative capabilities. Integrating DCA into our analysis quantified the clinical benefits and potential harm across various decision thresholds, underscoring the net benefit of the nomogram in clinical practice. This finding enhances the relevance of the model for guiding treatment decisions in elderly patients with urosepsis. Our calibration analysis revealed that the model tended to overestimate the risk at probabilities above 0.4, suggesting an optimistic bias in the model’s predictions, which could potentially influence the intensity of treatments administered. Conversely, it underestimated risk at probabilities between 0.15 and 0.40, which could lead to overly cautious clinical interventions that might not fully meet patients’ needs. Additionally, the calibration curve was smoother in the training set than in the validation set, indicating potential overfitting. These trends highlight the importance of integrating clinical judgment with the nomogram results and continuously evaluating the model to ensure effective treatment decisions in diverse settings. Despite these observed biases, the *P* value above 0.05 in our calibration tests suggests an acceptable overall fit, confirming the reliability in clinical applications. Validation through calibration plots, Hosmer–Lemeshow test, NRI, IDI, and DCA confirmed our model’s excellent ability to differentiate, calibrate, and validate the prediction of in-hospital mortality for the target patient population.

The prognostic factors related to sepsis have been extensively studied. Lactate levels, renal insufficiency, thrombocytopenia, pulmonary infections, UTI, and hyperthermia have been identified as risk factors for adverse sepsis outcomes [23–26], whereas higher levels of plasma albumin and IgG may provide protection [27, 28]. Current research on urosepsis in the elderly primarily focuses on its diagnosis, particularly the early identification and differentiation of subtle differences between tract infections and urosepsis. Advanced biomarkers and the use of machine learning techniques play specific roles in the early diagnosis of urosepsis and are being studied to improve diagnostic accuracy [29, 30]. However, research on the risk factors affecting outcomes in older patients with urosepsis is lacking. Our study identified GCS, WBC, RDW, and invasive ventilation as risk factors for elderly patients with urosepsis. These findings were used to establish a nomogram to predict the risk of in-hospital mortality in this patient population.

Among these variables, the OR for GCS was less than 1, indicating a negative association with in-hospital mortality in elderly patients with urosepsis. One study found a statistically significant difference in the GCS between survivors and non-survivors of urosepsis, with a higher GCS associated with better outcomes [31]. Another study discovered that the GCS was superior to other factors in predicting the prognosis of critically ill children with urosepsis [32]. The same relationship was observed in our study, which is consistent with the clinical outcomes. Patients with lower GCS scores exhibit impaired consciousness, potentially due to systemic inflammatory responses from infections, metabolic disorders, or direct brain injury [33]. In clinical practice, doctors must closely monitor the neurological status of patients with low GCS scores, promptly identify potential neurological complications, and consider early interventions such as surgical treatment of the infection source, antibiotics, and supportive care to mitigate the risk of long-term neurological damage [31]. Additionally, GCS scores can assist doctors in assessing patient responses to treatment and serve as a basis for adjusting treatment plans.

In our model, invasive ventilation carried the greatest weight, indicating that it was the most crucial indicator of in-hospital mortality among elderly individuals with urosepsis. A study conducted in France and Spain across 18 medical centers reported an early intubation rate of 24% and a cumulative intubation rate of 38% during the ICU stay, which were remarkably high [34]. Although mechanical ventilation provides essential respiratory support, it can cause a range of complications in elderly patients. Research has indicated that mechanical ventilation increases the possibility of complications such as lung injury, ventilator-associated pneumonia, and long-term dependency on mechanical ventilation in elderly patients [35]. Another study showed that elderly patients are more likely to develop UTI caused by extended spectrum beta lactamase (ESBL)-producing Escherichia. coli [36]. These complications increase the risk of in-hospital mortality in elderly patients with urosepsis. The need for invasive ventilation often signifies the severity of urosepsis, implying a significant deterioration in the patient’s physiological state. This is particularly pronounced in the elderly population, whose physiological and immune systems tend to become more fragile due to aging [37]. When elderly patients with urosepsis require invasive ventilation, it not only reflects severe impairment of their physiological functions but also indicates a higher risk of in-hospital mortality. Therefore, physicians should carefully consider the overall condition and prognosis of elderly patients when deciding whether to initiate invasive ventilation. For patients who have already received invasive ventilation, strict infection control measures should be implemented, weaning should be regularly assessed, and early rehabilitation training should be considered to promote overall recovery.

Our study also found that elevated RDW was associated with a higher rate of in-hospital mortality among elderly patients with urosepsis. Currently, the RDW is a significant predictor factor of human mortality. An increase in RDW could serve as an important biomarker for diagnosing urosepsis [4]. Another study confirmed that survivors of sepsis had significantly lower levels of RDW than non-survivors [38]. The underlying mechanism may involve inflammatory markers such as tumor necrosis factor-alpha, interleukin-6, and other pro-inflammatory cytokines, which inhibit the maturation process of red blood cells and increase their half-life, leading to elevated levels of RDW [39]. These findings suggest that RDW may be a simple and easily implemented prognostic marker for predicting sepsis outcomes and mortality. Therefore, patients with higher RDW levels should receive extra care.

The in-hospital mortality rate of elderly patients with urosepsis is high, making their clinical treatment more challenging. Various systems, such as the quick SOFA (qSOFA), SOFA, APACHE-II, and SAPS-II scores, are used for the clinical assessment of patients with sepsis [40]. These scoring systems, which have shown improvements in mortality differentiation, calibration, and predictive ability, are recommended to identify and predict the prognosis of patients with sepsis [41, 42]. To meet the demands of clinical practice and fully understand the progression of sepsis, numerous researchers have integrated various biomarkers to predict mortality in patients with sepsis [43, 44], with some studies combining biomarkers with scoring systems to do so. For example, Rijhwani et al. [45] found that combining biomarkers (lactate, C-reactive protein, procalcitonin) with the qSOFA score predicted the 28-day mortality of patients with sepsis better than using the qSOFA alone. However, to our knowledge, no comprehensive studies have been conducted on predictive model for the risk of in-hospital mortality among elderly patients with urosepsis. Therefore, we collected the clinical information of elderly patients diagnosed with urosepsis using the MIMIC-IV database. Logistic regression was used to determine the risk factors associated with in-hospital mortality, validate the predictive model, create a nomogram, and evaluate the effectiveness and calibration of the model. Our study indicates that the newly developed nomogram provides higher predictive accuracy for in-hospital mortality among elderly patients with urosepsis. These advancements are crucial as an accurate predictive model is essential for early intervention, efficient treatment strategies, and ultimately improving patient outcomes.

### Strengths and limitations

A key strength of this study is the use of MIMIC-IV, an extensive public database containing extensive information on critically ill patients. Furthermore, we constructed a nomogram to evaluate the risk of in-hospital mortality based on laboratory tests and complications upon admission in elderly patients with urosepsis, demonstrating the effectiveness of the model in a previously unaccomplished manner. However, this study has several limitations. First, this was a single-center study with no external validation despite the large sample size. Second, as this study was a retrospective secondary data analysis, a selection bias may inevitably be present. Third, missing data were addressed using multiple imputations, which might reduce the accuracy of the model. Fourth, as only four of the original 43 predictive indicators were retained for model construction, the small number of variables included in the predictive model may have limited its predictive efficiency. Finally, our study used a nomogram based on logistic regression. Advanced predictive models such as machine learning algorithms offer significant advantages in handling large datasets and uncovering non-linear relationships; therefore they have the potential to enhance predictive accuracy. Future research could explore integrating machine learning techniques to compare their effectiveness in similar clinical scenarios.

## Conclusions

In conclusion, the novel nomogram developed in this study, which includes GCS, WBC, RDW, and invasive ventilation, can accurately predict the in-hospital mortality rate of elderly ICU patients with urosepsis. Therapeutic strategies that address the factors considered in this model can improve in-hospital mortality rates.

## Data Availability

The datasets presented in the current study are avaliable in the MIMIC IV 2.2 database (https://physionet.org/content/mimiciv/2.2/).

## Abbreviations

UTI: Urinary tract infection
ICU: Intensive care unit
MIMIC: Medical Information Mart for Intensive Care
GCS: Glasgow coma scale
WBC: White blood cell
RDW: Red cell distribution width
ROC: Receiver operating characteristic
AUC: Area under the receiver operating characteristic curve
NRI: Net reclassification improvement
IDI: Integrated discrimination improvement
DCA: Decision curve analysis
qSOFA: quick Sequential organ failure assessment
SOFA: Sequential organ failure assessment
APACHE: Acute Physiology and Chronic Health Evaluation
SAPS: Simplified Acute Physiologic Score
ICD: International Classification of Diseases
LASSO: Least Absolute Shrinkage and Selection Operator
OR: Odds ratio
CI: Confidence interval
ESBL: extended spectrum beta lactamase
